# Platelet-leucocyte interactions drive MMP-mediated tissue damage in tuberculosis

**DOI:** 10.1371/journal.ppat.1014205

**Published:** 2026-05-11

**Authors:** Daniela E. Kirwan, Deborah L. W. Chong, Oscar Gayoso, Jorge Coronel, Maria-Cristina Loader, Cesar A. Ugarte-Gil, Lilia Cabrera, Ricardo C. Medrano, Kirk A. Taylor, Michael Emerson, Ivan Lozada-Requena, Julia Kutschenreuter, Mirko Zimic, Robert H. Gilman, Jon S. Friedland

**Affiliations:** 1 Institute for Infection & Immunity, City St. George’s, University of London, London, United Kingdom; 2 Department of Pulmonology, Hospital Nacional Cayetano Heredia, Lima, Peru; 3 Laboratorio de Investigación de Enfermedades Infecciosas: Mycobacterium, Laboratorios de Investigación y Desarrollo, Facultad de Ciencias e Ingeniería, Universidad Peruana Cayetano Heredia, Lima, Peru; 4 Instituto de Medicina Tropical Alexander von Humboldt, Universidad Peruana Cayetano Heredia, Lima, Peru; 5 Department of Epidemiology, School of Public and Population Health, University of Texas Medical Branch, Galveston, Texas, United States of America; 6 Asociación Benéfica Prisma, Lima, Peru; 7 Laboratorios de Investigación y Desarrollo, Facultad de Ciencias e Ingeniería, Universidad Peruana Cayetano Heredia, Lima, Peru; 8 Institute for Cardiovascular and Metabolic Research, School of Biological Science, University of Reading, Reading, United Kingdom; 9 National Heart and Lung Institute, Imperial College London, London, United Kingdom; 10 Laboratorio de Inmunología #108, Laboratorios de Investigación y Desarrollo, Facultad de Ciencias e Ingeniería, Universidad Peruana Cayetano Heredia, Lima, Peru; 11 Laboratorio de Bioinformática, Biología Molecular y Desarrollos Tecnológicos, Laboratorios de Investigación y Desarrollo, Facultad de Ciencias e Ingeniería, Universidad Peruana Cayetano Heredia, Lima, Peru; 12 Department of International Health, Johns Hopkins Bloomberg School of Public Health, Baltimore, Maryland, United States of America; New Jersey Medical School, UNITED STATES OF AMERICA

## Abstract

Tuberculosis causes inflammation and excess matrix metalloproteinase (MMP) activity which lead to tissue damage and adverse patient outcomes. Platelets are emerging as key drivers of inflammation, and platelet-leucocyte aggregate formation via interactions between platelet P-selectin and monocyte PSGL-1 receptors may regulate tissue destruction in tuberculosis. First, a platelet-monocyte co-culture model was utilised to assess platelet-leucocyte interactions. We then examined M.tb-infected and control lymph node tissue using immunofluorescence microscopy. Finally, we investigated tuberculosis patients (TB, n = 17), healthy controls (HC, n = 14), and patients undergoing bronchoscopy subsequently classified as TB (n = 10) or respiratory symptomatic (RS, n = 14). Whole blood was collected to quantify platelet aggregation using light transmission aggregometry, and platelet-monocyte aggregates (PMA), platelet-neutrophil aggregates (PNA), and platelet receptor expression using flow cytometry. In M.tb-infected monocytes, addition of platelets significantly increased secretion of MMP-1 and MMP-10 and upregulated *mmp1* gene expression 4.7-fold. MMP-1 secretion was also increased by addition of platelet-derived soluble factors, and by monocyte PSGL-1 receptor ligation. We observed abundant platelets in M.tb-infected lymph node tissue, localising to PSGL-1 receptors on monocytic cells, and this was not seen in M.tb-uninfected control tissue from patients with reactive hyperplasia or with lymphoma. *Ex vivo* platelet aggregation in response to stimulation with platelet agonist ADP (3µM, 10µM, and 30µM) was reduced in patients with TB versus HC. PMA were increased in TB and RS versus HC, while PNA were raised only in TB; platelet receptor expression was unchanged. Platelet P-selectin expression, PMA, and PNA correlated with each other but were independent of platelet expression of GPIIb/IIIa, indicating dissociation from thrombotic pathways. In summary, PSGL-1/P-selectin mediated platelet-leucocyte interactions drive inflammation and secretion of MMPs in pulmonary TB. This identifies platelets as important regulators of tissue-damaging inflammatory responses in tuberculosis. Targeting this pathway represents a potential host-directed therapeutic strategy in tuberculosis, and possibly in other lung diseases.

## Author summary

Each year, over 10 million people develop tuberculosis (TB) and around 1.25 million people die from the disease, often despite appropriate treatment. In patients with TB, enzymes called matrix metalloproteinases (MMPs) are released as part of the inflammatory response. These enzymes cause tissue damage, contributing to disease manifestations and deaths. Although platelets are increasingly known to be important in inflammation, we know very little about their role in TB. In this study we show that platelets, normally found in the blood, are present in tissues infected with *Mycobacterium tuberculosis* (M.tb). We also show that in TB patients platelets interact with immune cells through P-selectin/PSGL-1 receptor interactions to form platelet-leucocyte aggregates. This enhances immune cell activation and promotes release of MMPs. These interactions could be disrupted in TB patients by targeting platelet P-selectin or leucocyte PSGL-1 receptors using host-directed therapies. Such interventions may reduce platelet-driven inflammation in TB patients, limiting the release of tissue-damaging enzymes, and ultimately improve clinical outcomes. Similar approaches may also be useful in other fibrotic lung diseases.

## 1. Introduction

Post COVID-19, tuberculosis (TB) is once again the leading cause of death from a single infectious agent: in 2024 there were 10.7 million new cases of TB and 1.25 million deaths [1]. A significant proportion of deaths occur despite appropriate anti-TB treatment [2]. This is largely due to an excessive inflammatory immune response to the causative agent, *Mycobacterium tuberculosis* (M.tb), leading to secretion of tissue-degrading enzymes particularly matrix metalloproteinases (MMPs) [3]. This results in severe disease pathology and facilitates transmission [4]. Moreover, damaged tissue heals with fibrosis, so even successfully treated patients suffer long-term sequelae including impaired lung function and quality of life [5–7]. Platelets may be key drivers of tissue damage, but little is understood about their contribution to inflammation in TB.

Platelet function is not restricted to haemostasis and thrombosis, with platelets increasingly seen as important regulators of immune responses that arrive promptly at sites of infection, recruit and activate other immune cells, and secrete immunoregulatory molecules [8,9]. Platelet count is often raised in TB patients [10], markers of platelet activity are present [11], and platelet-associated gene transcripts are upregulated in their blood [12] and infected tissue [13,14]. High numbers of platelet-monocyte aggregates (PMA) have been observed in TB patients [15,16] which, as in other inflammatory conditions [17], may indicate active inflammation. Formation of platelet-leucocyte aggregates results from various mechanisms, including engagement between platelet P-selectin (CD62P) and leucocyte P-selectin glycoprotein ligand-1 (PSGL-1, CD162) receptors [18,19], but in TB such interactions have not been studied.

This study investigated platelet-leucocyte interactions in TB and the specific role of P-Selectin/PSGL-1 interactions in TB immunopathology using combined clinical and cellular approaches.

## 2. Results

### 2.1 Platelets increase MMP-1 secretion and gene expression from M.tb-stimulated human monocytes

**Fig 1:**
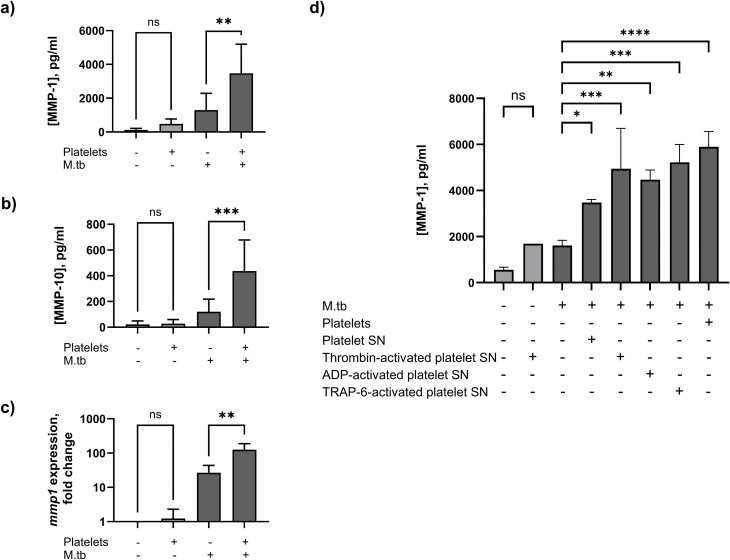
Platelets enhance MMP secretion and gene expression from M.tb-stimulated monocytes. **(a)** MMP-1 and **(b)** MMP-10 secretion increases from M.tb-infected monocytes co-cultured with platelets. **(c)** *mmp1* gene expression significantly increases in M.tb-infected monocytes co-cultured with platelets for 24h. **(d)** Supernatant (SN) from unstimulated platelets and from platelets stimulated by thrombin, ADP, or TRAP-6 increases secretion of MMP-1 from M.tb-infected monocytes. Graphs represent pooled data from seven (a,b) or four (c) separate experiments, each experiment performed in duplicate, or representative of two (d) experiments, performed in triplicate. Mean ± standard deviation (SD) are shown. Data compared with one-way ANOVA with Šídák’s correction for multiple comparisons; *p < 0.05, **p < 0.01, ***p < 0.001, ****p < 0.0001.

First, we examined the effect of platelets on MMP secretion and gene expression by M.tb-infected monocytes. Stimulating human monocytes with M.tb increased MMP-1 secretion from 124.8 ± 88.3 pg/ml to 1,298 ± 990 pg/ml and platelets increased this further to 3,479 ± 1,720 pg/ml (p = 0.0018, Fig 1a). Similarly, monocyte MMP-10 secretion increased from 21.5 ± 27.2 pg/ml to 119.9 ± 97.9 pg/ml when stimulated with M.tb, and to 436.3 ± 242.2 pg/ml with addition of platelets (p = 0.0008, Fig 1b). Increased MMP-1 secretion was driven by increased gene expression: M.tb stimulation increased monocyte *mmp1* expression 26.6-fold, and platelets increased this further to 125.4-fold compared to unstimulated monocytes (p = 0.0021, Fig 1c).

In healthy circulation, platelets are quiescent and become activated at sites of injury or infection. Thrombin is a potent physiological platelet agonist that cleaves protease-activated receptors (PARs) triggering rapid and irreversible platelet activation. In contrast, ADP is a moderate platelet agonist that primarily amplifies platelet responses. Thrombin receptor-activating peptide-6 (TRAP-6) mimics thrombin by selectively activating PAR-1 receptors without forming fibrin allowing controlled analysis of platelet function *in vitro*. To evaluate the contribution of platelet-secreted factors on monocyte function in TB, platelet supernatant or platelets were added to M.tb-stimulated monocytes. Platelet supernatant increased MMP-1 secretion from 1,607 ± 231.8 pg/ml to 3,479 ± 132.5 pg/ml (p = 0.018, Fig 1d). To better simulate *in vivo* platelet phenotype, we also added supernatant from platelets pre-activated with thrombin, ADP, or TRAP-6. This increased MMP-1 concentrations further, but concentrations were highest after addition of platelets themselves to M.tb-infected monocytes (5,897 ± 669.2 pg/ml, Fig 1d).

### 2.2 Platelet-induced MMP secretion in M.tb-stimulated monocytes is partly dependent on P-selectin binding to PSGL-1

**Fig 2:**
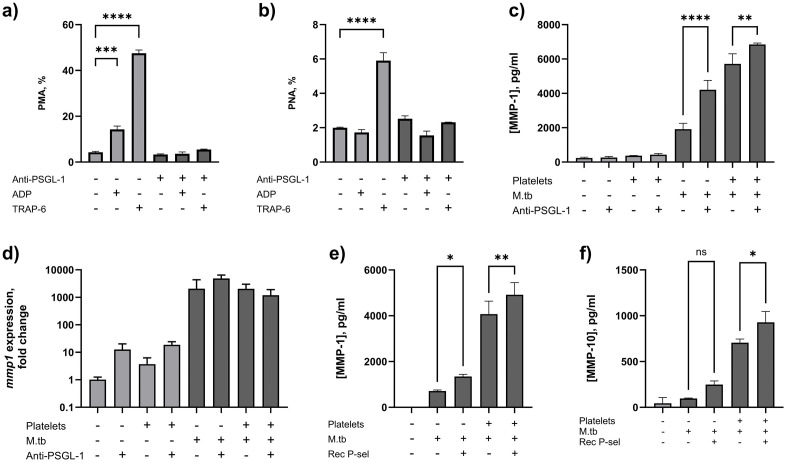
PSGL-1 receptor ligation inhibits agonist-induced platelet-leucocyte aggregation, and enhances MMP secretion by M.tb-infected monocytes in the presence or absence of platelets. Anti-PSGL-1 antibody inhibits ADP- and TRAP-6-induced **(a)** platelet-monocyte aggregation (PMA) and **(b)** platelet-neutrophil aggregation (PNA) in whole blood, by flow cytometry. **(c)** Ligation of the PSGL-1 receptor with anti-PSGL-1 antibody significantly increases secretion of MMP-1 by M.tb-infected monocytes both in the presence and absence of autologous platelets, but **(d)** does not affect *mmp1* gene expression. The addition of recombinant P-selectin (Rec P-sel) increases secretion of **(e)** MMP-1 and **(f)** MMP-10 from M.tb-infected monocytes. All experiments performed at least in duplicate. Bars represent mean + /- SD. Data compared using one-way ANOVA with Šídák’s correction for multiple comparisons. * p<0.05, **p < 0.01, ***p < 0.001, ****p < 0.0001.

Binding of anti-PSGL-1 monoclonal antibody (clone KPL1) mimics PSGL-1 stimulation with its physiological ligand, P-selectin [20–22]. Using flow cytometry, we demonstrated that anti-PSGL-1 antibody inhibits PMA formation in whole blood following stimulation with ADP and TRAP-6, and platelet-neutrophil aggregate (PNA) formation following stimulation with TRAP-6 (Fig 2a and 2b). Expression of P-selectin and GPIIb/IIIa receptors can be used as measures of platelet activation: following platelet stimulation P-selectin is translocated from alpha granules to the surface membrane, whereas GPIIb/IIIa, an integrin that mediates platelet aggregation and thrombus formation by binding fibrinogen, undergoes conformational change to its active form. In whole blood stimulated with ADP and TRAP-6 expression of platelet P-selectin and active GPIIb/IIIa were unchanged (S1 Fig), indicating absence of platelet activation.

In M.tb-infected human monocytes, PSGL-1 ligation increased MMP-1 secretion from 1,914 ± 343 pg/ml to 4,212 ± 545.2 pg/ml (p < 0.0001), and this significantly increased following addition of platelets (6,850 ± 80.94 pg/ml, p < 0.0001, Fig 2c). There was no difference in *mmp1* gene expression (Fig 2d). Next, 1µg/ml recombinant P-selectin was added to M.tb-infected monocytes. MMP-1 secretion increased from 713.7 ± 45.4 pg/ml to 1,346 ± 94.6 pg/ml (p = 0.044), and, with addition of platelets, from 4,072 ± 568 pg/ml to 4,919 ± 530.1 pg/ml (p = 0.0076, Fig 2e). Findings were similar for MMP-10 secretion (Fig 2f).

In summary, platelets increase MMP-1 and MMP-10 secretion and gene expression in M.tb-stimulated monocytes through a combination of contact-dependent signalling mediated through P-selectin/PSGL-1 receptors, and stimulation by factors released by activated platelets. We next sought to establish whether this occurred in TB patients.

### 2.3 Platelets localise to PSGL-1 receptors in M.tb-infected patient tissues

**Fig 3:**
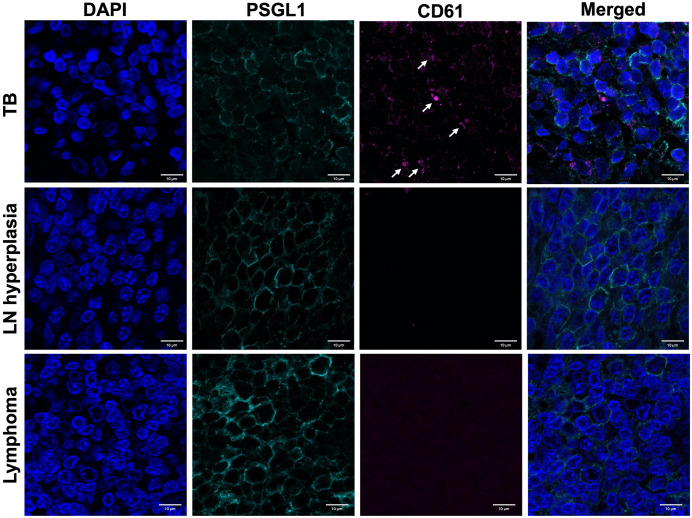
Platelets are present in M.tb-infected lymph node tissue. CD61+ (pink) platelet aggregates (white arrows) localise to the surface of PSGL1+ (green) cells (with blue DAPI nuclear co-staining) in lymph node tissue from TB (upper panel) but not in control patients with lymph node hyperplasia (middle panel) or lymphoma (lower panel), detected by immunofluorescence staining. Tissue samples from 3 patients per group were stained and examined using microscopy. Representative confocal microscopy images are shown, and size is denoted by the scale bar (10μm).

We investigated the relationship between platelets and PSGL-1 in M.tb-infected and uninfected human lymph node tissue. P-selectin glycoprotein ligand-1 (PSGL-1) is a regulator of monocyte function and migration [23] that binds with platelet P-selectin with resultant downstream signal transduction [24]. Using immunofluorescence staining, we examined tissue from HIV-negative patients with culture- and histologically-confirmed TB lymphadenitis and from control patients with reactive hyperplasia and with lymphoma. Platelets, identified by CD61 expression, were clearly visible in M.tb-infected but not uninfected control tissue. In infected tissue, platelets localised to areas high in PSGL-1 receptor expression on monocytic cell surfaces (Fig 3).

### 2.4 Clinical cohort

We enrolled 17 patients with sputum smear-positive pulmonary TB, 14 healthy controls, and 25 patients undergoing bronchoscopy of whom ten were classified as having TB and 14 as TB-negative respiratory symptomatic controls; one was excluded from analysis due to concurrent pleural TB. Demographic and clinical data are presented in Table 1. TB patients were younger than healthy controls (median age 28 vs. 45 years, p = 0.036) and respiratory symptomatic patients (median age 62 years, p < 0.0001). There were no sex differences between groups. Median BMI was lower in TB patients than healthy controls (23.1 vs. 27.1, p = 0.0024) and respiratory symptomatic controls (24.8, p = 0.041).

**Table 1:** Demographics of all patients enrolled to the study.

	Pulmonary TB patients (N = 27)¥	Respiratory symptomatic patients (N = 14)	Healthy controls (N = 14)
Age, median (IQR)	28 (22-44)	62 (50-73)	45 (33-53)
No. of males (%)	14 (51.9%)	5 (35.7%)	4 (28.6%)
BMI, median (IQR)*	23.1 (20.7-24.8)	24.8 (22.8-30.7)	27.1 (25.7-27.8)
**Site of recruitment**			
Pulmonology department, HNCH (%)	17 (63.0%)	13 (92.9%)	14 (100%)
Emergency department, HNCH (%)	1 (7.1%)	0	0
Hospital inpatient, HNCH (%)	1 (7.1%)	1 (7.1%)	0
Health centre (%)	8 (29.6%)	0	0
**Smoking and alcohol history**			
Current or ex-smoker (%)	12 (44.4%)	3 (21.4%)	0
Drinks alcohol at least once per month	18 (66.7%)	8 (57.1%)	9 (64.3%)
**TB contact**			
History of family member with TB (%)	8 (50%)	5 (29.4%)	2 (14.3%)
Other known TB contact (%)	3 (18.8%)	1 (5.9%)	3 (21.4%)
**Past medical history**			
Lung disease (asthma/COPD) (%)	5 (18.5%)	1 (5.9%)	1 (5.9%)
Heart disease (%)	0	0	0
Cerebrovascular disease (%)	0	0	0
Diabetes	3 (18.8%)	0	11 (5.9%)
**Antibiotics**			
Taking antibiotics (%)	7 (25.9%)	2 (14.3%)	0
Anti-TB treatment (%)	7 (25.9%)	0	0
Days on anti-TB therapy, median (IQR)	2 (1.5-4.5)	N/A	N/A

¥*data other than basic demographics not available for one patient from the pulmonary TB group*.

*data missing from 2 pulmonary TB patients and 1 healthy control.

*IQR: Interquartile range. HNCH: Hospital Nacional Cayetano Heredia. COPD: chronic obstructive pulmonary disease*.

Of the ten bronchoscopy patients diagnosed with TB, seven were culture-positive including five who were also acid-fast bacilli (AFB) positive on microscopy. Three patients were negative according to all microbiological tests and were diagnosed and treated based on clinical and radiological findings (S1 Table). TB-negative bronchoscopy patients were diagnosed with hypersensitivity pneumonitis (n = 3), lung cancer (n = 2), bacterial pneumonia (n = 2), bronchiectasis (n = 1), COPD exacerbation (n = 1), chronic bronchitis (n = 1), chronic inflammation secondary to gastric reflux (n = 1), interstitial lung disease (n = 1), or no diagnosis reached (n = 2).

### 2.5 Agonist-induced platelet aggregation is decreased in TB patients

**Fig 4:**
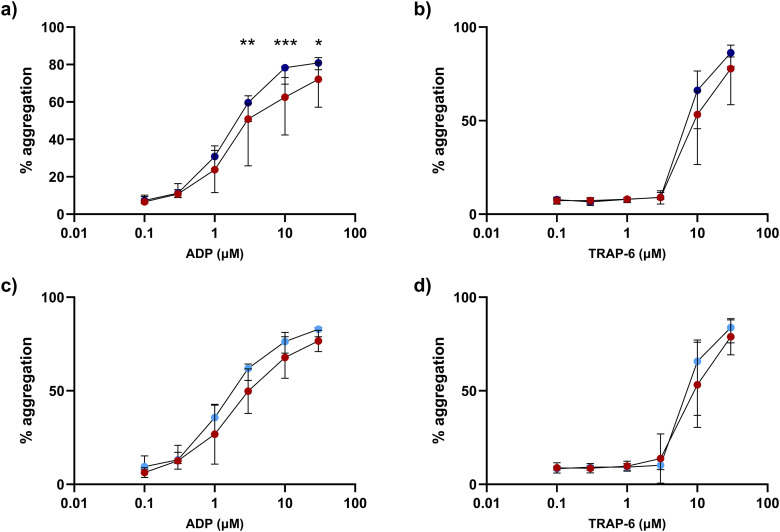
Platelet aggregation is reduced in patients with TB compared to TB-negative controls. Data show maximal platelet aggregation in response to stimulation with increasing concentrations of **(a,c)** ADP or **(b,d)** TRAP-6. Platelet aggregation was reduced in patients with newly diagnosed smear-positive pulmonary TB (red, n = 17) compared to healthy controls (dark blue, n = 14) in response to higher concentrations of **(a)** ADP but not **(b)** TRAP-6. In patients who underwent a bronchoscopy and were subsequently diagnosed with TB (red, n = 10) compared to bronchoscopy patients who were not diagnosed with TB (light blue, n = 14) there was a trend towards reduced agonist-induced maximal platelet aggregation in patients who were diagnosed with TB **(c,d)**. Data points represent median value and error bars show interquartile ranges. Data compared using a two-way ANOVA with Šídák’s correction for multiple comparisons. * p < 0.05, ** p < 0.01, *** p < 0.001.

In sputum smear-positive TB patients (n = 17) versus healthy controls (n = 14), platelet aggregation was significantly reduced following stimulation with ADP at 3µM (44.8% vs 57.5%, p = 0.0079), 10µmM (59.7% vs 75.6%, p = 0.0005), and 30µM (69.0% vs 79.9%, p = 0.33, Fig 4a). No difference in response to TRAP-6 stimulation was seen (Fig 4b). A similar but non-significant trend was observed in bronchoscopy patients with TB (n = 10) compared to respiratory symptomatic controls (n = 14, Fig 4c and 4d).

**Fig 5:**
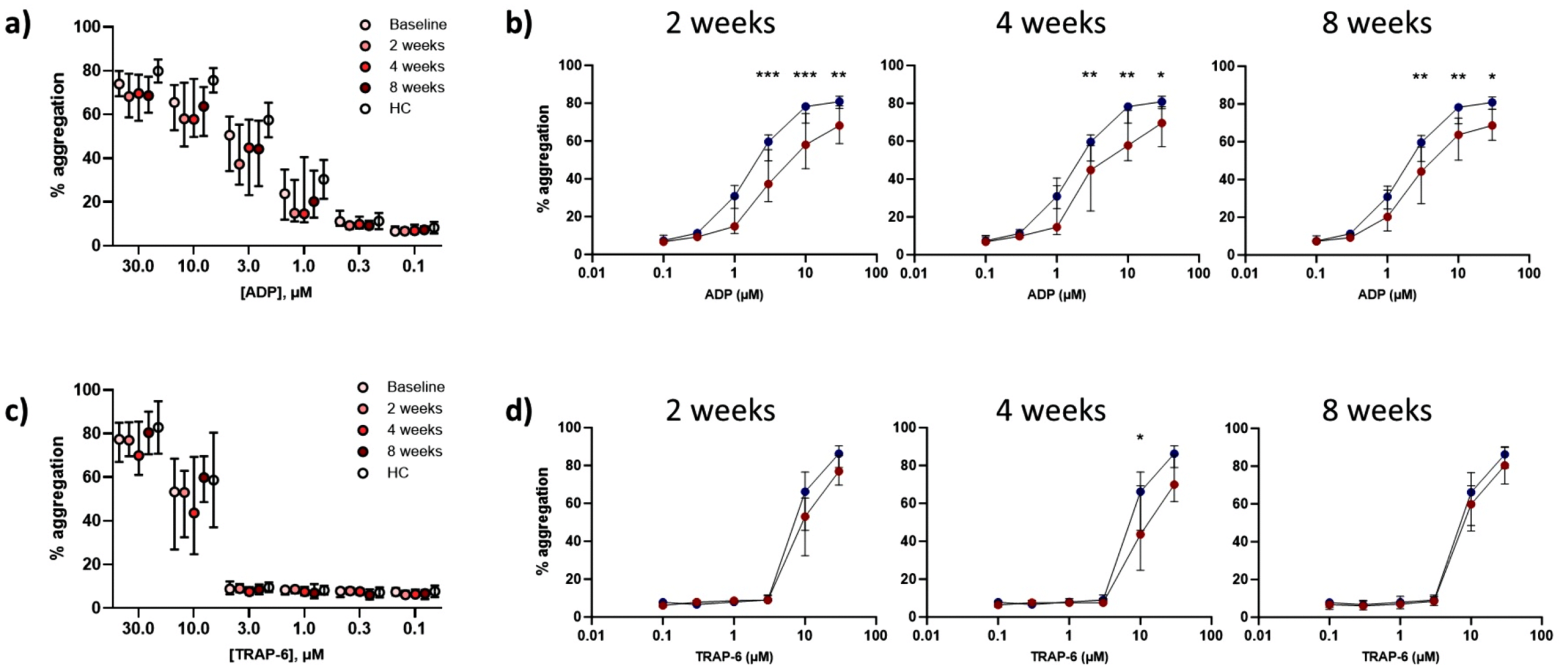
Changes in platelet aggregation in patients with TB normalise over time with treatment. Maximal agonist-induced aggregation shown over time in response to **(a)** ADP and **(c)** TRAP-6 at diagnosis (baseline, light pink, n = 27) and after 2 weeks (n = 10), 4 weeks (n = 10), and 8 weeks (n = 6); later timepoints indicated by increasingly darker red circles, healthy controls (HC) included for comparison (n = 14, open circles). A trend towards further reduction in aggregation over time is seen, with a normalisation by 8 weeks. Maximal agonist-induced aggregation in response to **(b)** ADP and **(d)** TRAP-6 in TB patients (red, n = 10) after 2 weeks, 4 weeks, or 8 weeks of anti-TB treatment compared to healthy controls (blue, n = 14). **(b)** Platelet aggregation was significantly reduced in TB patients in response to higher concentrations of ADP. **(d)** Platelet aggregation was reduced in TB patients after four weeks of anti-TB treatment following stimulation with 10µM TRAP-6, but was no different by 8 weeks. Data points represent median value and error bars show interquartile ranges. Data compared using a two-way ANOVA with Šídák’s correction for multiple comparisons. *p < 0.05, **p < 0.01, ***p < 0.001.

Of 27 patients with TB, follow-up data were available after 2 (n = 10), 4 (n = 10), and 8 weeks of treatment (n = 6). In TB patients versus healthy controls, diminished aggregation response to ADP was greatest after two weeks and remained lower after 8 weeks (Fig 5a and 5b). In response to TRAP-6, platelet aggregation was reduced in TB patients after 4 weeks only (45.3% vs 58.7%, p = 0.019, Fig 5c and 5d).

### 2.6 Increased patient platelet-neutrophil aggregates are TB-specific

**Fig 6:**
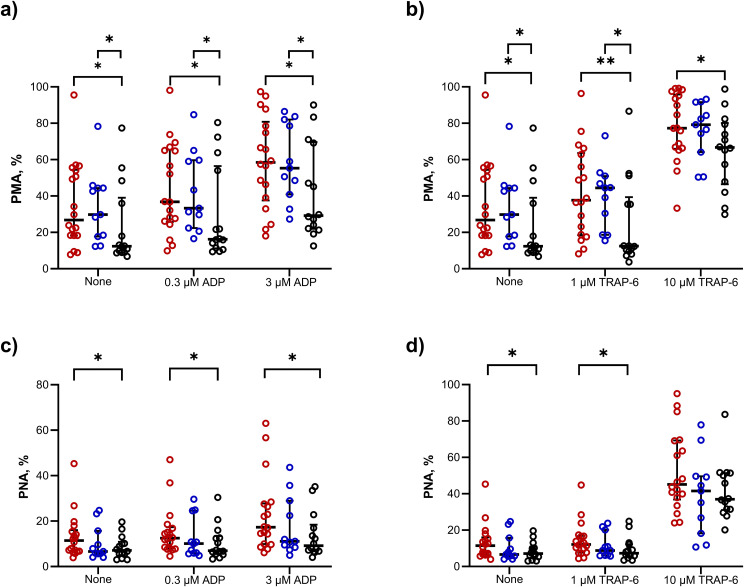
PMA are increased in TB patients and non-TB symptomatic patients compared to healthy controls, whereas PNA are increased in TB patients only. Graphs show the percentage of platelet-monocyte aggregates (PMA) or platelet-neutrophil aggregates (PNA) in unstimulated whole blood and in the presence of increasing **(a,c)** ADP or **(b,d)** TRAP-6 stimulation from TB patients (red circles), non-TB respiratory symptomatic controls (blue circles), or healthy controls (black circles). Data presented as median + /- IQR. Results compared using one-tailed Mann-Whitney U test. *p < 0.05, **p < 0.01.

Next, we showed that PMA were significantly increased in TB patients versus healthy controls (26.8% vs. 12.4%, p = 0.03) and in respiratory symptomatic patients (29.8%) versus healthy controls (p = 0.029), but there was no difference between TB and respiratory symptomatic patients (Fig 6a and 6b). Following stimulation with ADP or TRAP-6, PMA remained elevated in TB patients and respiratory symptomatic patients versus healthy controls (Fig 6a and 6b).

In contrast, PNA were raised in TB patients versus healthy controls (11.5% vs 7.12%, p = 0.039), but not respiratory symptomatic patients versus control subjects (6.7%, p = 0.36, Fig 6c and 6d). PNA were also elevated in TB patients versus healthy controls following stimulation with ADP and 1µM TRAP-6 (Fig 6c and 6d).

Flow cytometry data of PMA or PNA from TB patients at baseline (n = 16) and after 2 (n = 7), 4 (n = 8), and 8 weeks (n = 4) of anti-TB treatment were compared to healthy controls (n = 14). PMA and PNA were not elevated at any timepoint beyond initiation of anti-mycobacterial therapy (S2 Fig).

### 2.7 TB does not affect expression of surface receptors on circulating platelets

**Fig 7:**
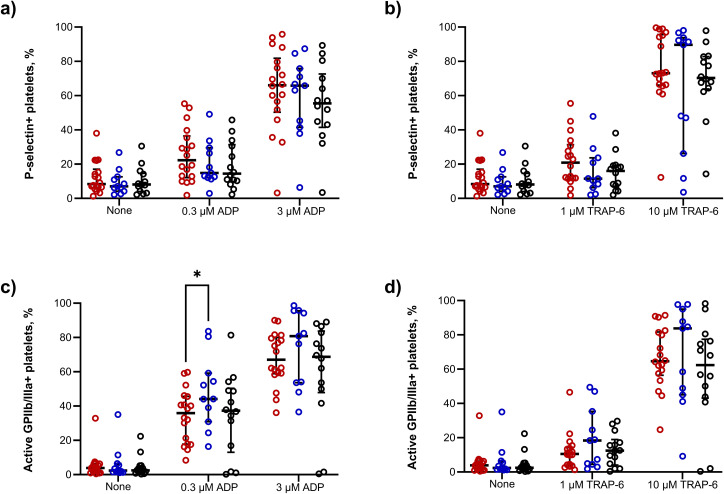
There is no difference in expression of P-selectin or active GPIIb/IIIa receptors on CD42b+ platelets in the whole blood of patients with TB compared to respiratory symptomatic patients or healthy controls. The percentage of CD42b+ platelets positive for P-selectin or active GPIIb/IIIa receptor was not significantly different in unstimulated whole blood, or in the presence of ADP **(a,c)** or TRAP-6 **(b,d)**, from patients with TB (red circles), symptomatic controls (blue circles), or healthy controls (black circles). Data presented as median + /- interquartile range. Comparisons with one-tailed Mann-Whitney U test. *p < 0.05.

We then compared the levels of platelet activation and of responsiveness to external stimuli across the patient groups. There was no difference in resting platelet P-selectin expression between TB patients, symptomatic controls, and healthy controls (8.36%, 7.02%, and 8.13% respectively, Fig 7a). Stimulation with ADP and TRAP-6 led to dose-dependent increases in platelet P-selectin expression, but there was no difference between patient groups (Fig 7a and 7b). Similarly, active GPIIb/IIIa expression on unstimulated platelets was unchanged (Fig 7c). In the presence of 0.3µM ADP, active GPIIb/IIIa expression was increased in respiratory symptomatic controls versus TB patients (Fig 7c). Stimulation of patient platelets with increasing concentrations of TRAP-6 increased active GPIIb/IIIa expression, but with no significant difference between patient groups (Fig 7d).

### 2.8 Platelet-leucocyte aggregation correlates with platelet P-selectin, but not active GPIIb/IIIa receptor expression

**Fig 8:**
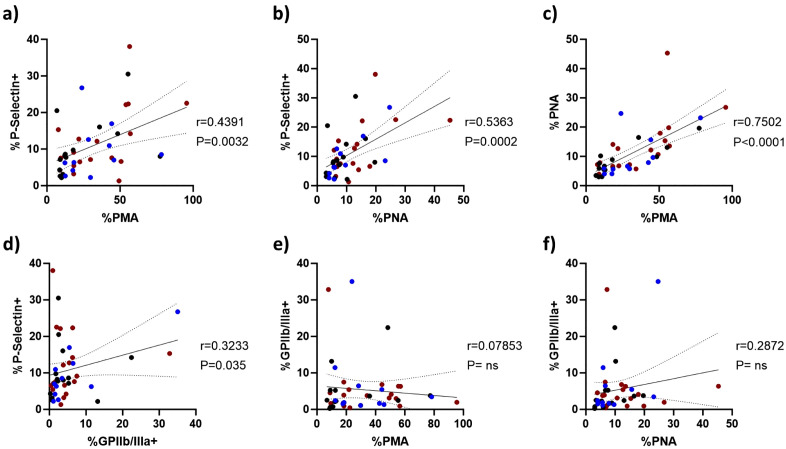
Platelet-leucocyte aggregation correlates with surface platelet P-Selectin, but not GPIIb/IIIa expression. PMA, PNA, platelet P-selectin and active GPIIb/IIIa expression were correlated in unstimulated TB platelets from patients (red), respiratory symptomatic patients (blue) and healthy controls (black). **(a)** PMA correlated positively with platelet P-selectin expression. **(b)** There was also strong positive correlation between PNA and platelet P-selectin expression, and **(c)** PMA and PNA correlated with each other (p < 0.0001). **(d)** Platelet surface expression of active GPIIb/IIIa expression correlated with P-selectin expression, but not with **(e)** PMA or **(f)** PNA. r = spearman’s coefficient of correlation. P = p-value.

To explore potential mechanistic relationships, we examined whether platelet receptor expression correlated with PMA and PNA formation. In unstimulated blood, platelet P-selectin expression correlated strongly with PMA (r = 0.44, p = 0.0032, Fig 8a) and PNA (r = 0.54, p = 0.0002, Fig 8b), and these with each other (r = 0.75, p < 0.0001, Fig 8c). P-selectin and active GPIIb/IIIa expression significantly correlated as expected since both increase upon platelet activation (r = 0.32, p = 0.035, Fig 8d). In contrast, platelet active GPIIb/IIIa expression correlated poorly with either PMA or PNA (Fig 8e and 8f). This is consistent with P-selectin’s role in platelet-leucocyte aggregate formation, whereas GPIIb/IIIa independently mediates platelet aggregation and thrombus formation via fibrinogen binding.

Following stimulation with ADP or TRAP-6, strong correlations were seen between PMA, PNA, and platelet P-selectin expression (S3–S5 Figs). Significant correlations between PMA, PNA, and platelet P-selectin with active GPIIb/IIIa expression were observed following stimulation with higher concentrations of ADP and TRAP-6 only (S6–S8 Figs).

## 3. Discussion

This study provides compelling cellular and clinical evidence for the importance of platelets in innate immune responses and MMP secretion in TB. First, using a co-culture model we confirm that platelets augment MMP-1 and MMP-10 secretion and gene expression in M.tb-infected monocytes [25]. We then identify for the first time that P-selectin/PSGL-1 receptor interactions regulate enhanced MMP responses, but contact-independent mechanisms must also be involved as platelet supernatant also increases monocyte MMP secretion. Platelet supernatant contains soluble P-selectin released from platelets which may be acting on PSGL-1 receptors [26], and platelet degranulation was recently identified in a proteomics analysis study as key biological processes in pulmonary TB [27].

We next assessed platelet activity and function in TB patients. We identified that platelets are present in M.tb-infected tissue, which is consistent with previous data [13,25]. We also showed that platelets localise to monocytic cells highly expressing PSGL-1 receptors. We next demonstrated decreased platelet aggregation responses in TB patients that became more pronounced in the first few weeks of treatment and remained low after 8 weeks. PNA were increased in TB patients only, whereas PMA were raised in both TB patients and respiratory symptomatic controls. Surface receptor expression on circulating platelets was similar across groups, although platelet expression of P-selectin, important in inflammation, strongly correlated with PMA and PNA, whereas correlation was poor with platelet GPIIb/IIIa receptors which are integral to clotting pathways. Together, these data demonstrate for the first time the key role of P-selectin/PSGL-1 receptor interactions in platelet-driven monocyte-derived tissue destruction in TB.

Enhanced monocyte MMP secretion following PSGL-1 stimulation was greater when platelets were present, indicative of additional mechanisms that enhance PSGL-1-driven effects. There may be interactions with other processes known to reinforce platelet-monocyte binding, including engagement between platelet CD40L and monocyte CD40 receptors [28] or stimulation of monocyte TLT-1 receptors [16]. TREM-like transcript 1 (TLT-1) receptor, like P-selectin, is stored in platelet alpha granules and translocates to the surface upon activation [29], and blocking monocyte TLT-1 receptor ligands reduces PMA formation [16]. Further work is needed to establish how these processes are linked, and which receptors are most effectively targeted to block subsequent monocyte activation.

We found that PMA and PNA are raised in TB patients compared to healthy controls [15,16] and normalise over time. We also studied heterogenous patients with non-TB lung disease, in whom PMA but not PNA were also elevated. This key finding suggests that elevated PMA occurs during a generalised systemic inflammatory response, whereas platelet-neutrophil interactions are relatively specific to TB. Neutrophils exert functions important in TB, including NET formation and MMP secretion [30]. In human lungs challenged with inhaled LPS, aspirin inhibited neutrophil influx and secretion of MMP-8 and MMP-9 [31]. PNA are implicated in inflammation and tissue damage in abdominal sepsis [32], although they have never previously been studied in TB. Our novel data suggest that interrupting platelet-neutrophil interactions may effectively control neutrophil-derived inflammation in pulmonary TB.

The mechanisms driving increased PMA in TB patients are unclear, including whether this occurs due to platelet and/or monocyte activation. P-selectin (CD62P) receptors reside in platelet granule membranes and externalise to the outer membrane during degranulation. The GPIIb/IIIa complex on the platelet surface undergoes conformational change upon platelet activation and the active form can be detected by mouse monoclonal antibody PAC-1 [33]. We found no change in expression of either receptor in circulating platelets. This suggests that platelet-monocyte aggregation in TB may be driven primarily by activated monocytes, or may reflect removal of activated platelets from the circulation. Our findings are consistent with published data [15], whereas others have found increased platelet activation in TB [13,16]: such divergence may reflect methodological differences, particularly as methods for assessing platelet activation are not standardised.

Our data clearly show decreased platelet aggregation in TB, and when viewed alongside the strong correlations linking PMA, PNA, and P-selectin expression, but not active GPIIb/IIIa expression, this supports a pro-inflammatory but not pro-thrombotic platelet phenotype in tuberculosis. Platelets differentially respond to discrete stimuli by engaging diverse intracellular signalling pathways [34]. During infection, they detect pathogen-associated molecular patterns (PAMPs) via surface receptors, triggering pathogen-specific functional responses that differ significantly across different infections. For example, in bacterial sepsis platelet activation leads to coagulopathy, which is closely integrated with innate inflammatory responses in a process termed thromboinflammation [35]. In contrast, in Dengue virus infection viral protein structures stimulate ADP-mediated platelet activation leading to increased aggregation and platelet dysfunction, resulting in thrombocytopaenia and haemorrhage [36]. Our findings have important implications for understanding platelet-driven pathology in TB and other infectious diseases, and for the development of targeted therapies.

A limitation of our clinical study was the small sample sizes with limited follow-up data due to diagnosed patients being transferred to their local health centres for ongoing treatment and the onset of the SARS-CoV-2 pandemic. Future studies are needed to investigate the effects of platelet activity on long-term outcomes in TB patients. Many TB survivors are left with lifelong complications, and TGF-β has been identified as a possible biomarker of lung fibrosis in post-TB lung disease [37]. Given that a significant proportion of circulating TGF-β originates from platelets [38] it is plausible that platelets are responsible for delivering high concentrations to granulomas, providing yet another incentive to better understand, and control, platelet activity in tuberculosis.

In summary, this study identifies a new and important role for PSGL-1/P-selectin driven platelet-leucocyte interactions in tissue-damaging inflammatory responses in pulmonary TB. The data suggest a unique platelet functional profile in TB patients, with reduced platelet aggregation and heightened inflammatory effects. Additionally, we have identified a central role for platelet-neutrophil interactions in driving TB immunopathology. Targeting platelet-dependent tissue destruction in TB is a potential approach to developing host-directed therapy that may improve outcomes in these patients and in others with diseases characterised by tissue damage.

## 4. Materials and methods

### 4.1 Ethics statement

The cellular work had ethical approval from St George’s, University of London (REC 18/NW/0193). The clinical study was approved by the Peruvian Ministry of Health (Dirección de Redes Integradas de Salud Lima Norte) and Institutional Review Boards at the Universidad Peruana Cayetano Heredia (SIDISI 102760), Hospital Nacional Cayetano Heredia (107–018), and the Asociación Benéfica PRISMA. The study was registered at the PRISA repository at the Peruvian National Institute of Health (INS) following local regulations (Code EI0000000527). Written informed consent was obtained from all patients.

### 4.2 M.tb culture

Work involving M.tb was performed in a Category 3 laboratory. M.tb H37Rv Pasteur was incubated in Middlebrook 7H9 medium supplemented with 0.2% glycerol, 0.02% Tween 80, and 10% OADC enrichment medium (BD Diagnostics, Oxford, UK) at 37°C, agitated at 120 rotations per minute (RPM), and sub-cultured weekly. Monocytes were infected by adding M.tb in the mid-log growth phase at a multiplicity of infection (MOI) of 1 (1x10^8^ – 2x10^8^ colony forming units (CFU)/ml) or control media [39].

### 4.3 Platelet-monocyte co-culture

Monocytes and platelets isolated from whole blood from consenting healthy volunteers were co-cultured as described [25]. Volunteers were asymptomatic staff working at City St George’s, University of London with no history of contact with tuberculosis. IGRA testing was not performed, however rates of positivity are low (<10%) in this population. Platelet:monocyte ratio used in experiments was 25:1 based on optimisation studies. M.tb or control media was added, cells were incubated for 24h at 37°C, then cell supernatants filter-sterilized and stored at -20°C [40]. Immediately cells were washed, lysed, and stored at -80°C.

To prepare platelet supernatant, platelets resuspended at 2.5x10^8^ cells/ml were incubated for 10min with or without 0.5U/ml thrombin, 10µM TRAP-6, (both platelet protein activating receptor 1 (PAR1) agonists), or 10µM ADP (platelet P2Y12 receptor agonist). Suspensions were centrifuged at 10,000*g* for 1min, then filtered to remove residual platelets.

Anti-PSGL-1 antibody (clone KPL1, 10µg/ml) was added to monocytes for 1h prior to addition of platelets. Recombinant P-selectin, platelet supernatant, activated platelet supernatant (1:4 final volume), or platelets (1.25x10^8^ cells/ml) were added to monocytes with no additional incubation time.

### 4.4 Enzyme-linked immunosorbent assay (ELISA)

MMP concentrations were quantified using the Duoset ELISA Development System (R&D Systems, Abingdon, UK) according to manufacturer’s instructions.

### 4.5 RNA extraction, cDNA synthesis, and RT-PCR

RNA was extracted using the Direct-zol RNA Miniprep Kit (ZymoResearch, Irvine, California, USA). Quantity and purity were evaluated by spectrophotometry (Nanodrop ND 1000, Thermo Fisher Scientific). cDNA was synthesised from RNA using the Quantitect Reverse Transcription Kit (Qiagen, Manchester, UK) then diluted to a total volume of 200µl in nuclease-free water. 15µl master-mix (Brilliant II (Agilent Technologies, USA), primer and probes for the gene of interest, and reference gene 18S rRNA [13,14]) and 10µl samples or standards were added to a 96-well plate and analysed using a CFX Connect Real-Time System Instrument (Bio-Rad Laboratories, UK) using a thermal profile of 95°C for 10min followed by 40 cycles of 95°C for 30s then 60°C for 1min.

### 4.6 Immunofluorescence confocal microscopy

Lymph node tissue was examined using immunofluorescence and microscopy for evidence of platelet activity. Lymph node tissue samples were obtained from a prior cohort of consenting patients. Briefly, adult patients in Lima, Peru with lymphadenopathy requiring diagnostic tissue sampling were prospectively recruited, and were later defined as TB cases or controls based on microbiological and histological results [41]. All patients were HIV-negative. Tissue from TB cases were M.tb culture-positive and demonstrated caseous granulomatous formation on histopathological examination. Control tissue samples were AFB smear- and culture-negative and had no evidence of TB infection on histology and were diagnosed with either reactive lymphadenopathy or lymphoma based on histopathological examination. Tissue was stained with rabbit anti-CD61 (1µg/ml, clone 2f2, Origene, USA) and mouse anti-PSGL1 (5µg/ml, clone KPL-1, Merck, UK) antibodies overnight at 4°C. Staining was developed using anti-mouse IgG Alexa Fluor-488 and anti-rabbit IgG Alexa Fluor-555 secondary antibodies and DAPI nuclear co-staining (all from Thermo Fisher Scientific, UK). Sections were mounted and images taken using a Nikon A1R confocal microscope.

### 4.7 Clinical study

Participants were prospectively recruited from the Hospital Nacional Cayetano Heredia (HNCH) or Peruvian National TB Programme clinics. Patients with newly diagnosed smear-positive pulmonary TB and healthy controls, identified from relatives of outpatients attending the general respiratory clinic at HNCH, were recruited in a case-control design, age- and sex-matched. A cohort of patients undergoing bronchoscopy was also recruited and patients retrospectively defined as TB or non-TB respiratory symptomatic controls once microbiological results became available. Inclusion criteria included written informed consent and age ≥ 18 years. Exclusion criteria included ≥7 days TB treatment, HIV infection, taking anti-platelet or immunosuppressive medications, known platelet or thrombo-embolic disease, malignancy, or pregnancy. HIV status was self-reported; where status was unknown patients were offered testing, in line with national policy.

Clinical and demographic information were collected using standardised questionnaires. Pulmonary TB patients provided sputum, and bronchoalveolar lavage fluid (BALF) was obtained during routine bronchoscopy procedures. Auramine microscopy and mycobacterial culture using the MODS method [42] were performed at Universidad Peruana Cayetano Heredia (UPCH). Blood samples were transported immediately to UPCH for light transmission aggregometry and flow cytometry. Participants with TB underwent prospective follow-up at 2, 4, and 8 weeks, and 6 months after treatment initiation, comprising a follow-up questionnaire and repeat blood sampling.

### 4.8 Platelet light transmission aggregometry

Whole blood collected in 10% sodium citrate (BD Vacutainer Sodium Citrate tubes) was centrifuged for 15min at 150*g* to obtain platelet rich plasma (PRP). This was added to serial dilutions of ADP or TRAP-6 or control wells in a 96-well plate, immediately inserted into a TECAN Sunrise plate reader, and absorbance read every 20s at 595nm at 37°C with shaking for 40 cycles.

### 4.9 Flow cytometry

25µl whole blood taken in 10% sodium citrate was incubated with binding buffer (1% w/v BSA), antibodies against CD14, CD42b, P-selectin (CD62P), and active GPIIb/IIIa (all from BD Pharminogen), with or without ADP or TRAP-6, at a final volume of 100µl for 20min. 500µl 1x eBioScience 1-step fix/lysis solution (Thermo Fisher Scientific) was added for 15min then 1ml PBS was added. Samples were stored at 4°C until analysis, within 72h. Compensation across channels was performed for each fluorophore combination, and false-positive aggregates minimized using slow flow rates. Data were acquired using a BD FACSCalibur flow cytometer (BD Biosciences), Peru, or a CytoFLEX S analyser (Beckman Coulter Life Sciences, UK), UK. Platelet P-selectin and active GPIIb/IIIa receptor expression, PMA, and PNA were quantified. Analysis was performed using FlowJo v10 software (BD Life Sciences).

For experiments investigating PSGL-1/P-selectin interactions, whole blood from healthy volunteers was incubated with 10µg/ml anti-PSGL-1 or anti-P-selectin antibody or PBS control for 10min, then processed as above.

### 4.10 Statistical analysis

Data were attained in Excel and analysed using GraphPad PRISM v9.1.2 (GraphPad, Boston, Massachusetts, USA). RT-PCR data were analysed using the ΔΔCT method [43] to generate fold change. Continuous variables were compared using *t*-tests or Mann-Whitney U tests, and multiple observations compared using one- or two-way ANOVA with Šídák’s correction for multiple comparisons, or Kruskall-Wallis test with Dunn’s *post hoc* correction for multiple comparisons. Correlations were assessed using Spearman’s test. p-values <0.05 were taken as significant.

## Supporting information

S1 Table Results of bronchoscopy report, radiological and microbiological investigations, and final diagnosis given to patients who underwent bronchoscopy. (DOCX)

S1 Fig PSGL-1 receptor ligation does not affect agonist-induced platelet expression of P-selectin or GPIIb/IIIa. (a) Platelet expression of P-selectin receptors was increased by stimulation with TRAP-6 but not ADP. This was not affected by addition of anti-PSGL-1 antibody. (b) Platelet expression of GBIIb/IIIa receptors was upregulated by stimulation with ADP and TRAP-6, and this was not affected by addition of anti-PSGL-1 antibody. Experiment performed in duplicate. Bars represent mean + /- SD. Data compared using one-way ANOVA with Šídák’s correction for multiple comparisons.
(TIF)

S2 Fig PMA and PNA normalise with TB treatment. Graphs show the percentage of platelet-monocyte aggregates (PMA) in whole blood from TB patients (green circles) vs healthy controls (black circles) using (a) unstimulated whole blood and following stimulation with (b) 0.3µM or (c) 3µM ADP or (d) 1µM or (e) 10µM TRAP-6. Percentage of platelet-neutrophil aggregates (PNA) in whole blood from TB patients (blue circles) vs healthy controls (black circles) using (f) unstimulated whole blood and following stimulation with (g) 0.3µM or (h) 3µM ADP or (i) 1µM or (j) 10µM TRAP-6. Data presented as median + /- IQR. Results compared using one-tailed Mann-Whitney U tests. *p < 0.05, **p < 0.01.
(TIF)

S3 Fig Correlation between platelet-monocyte aggregation (PMA) and platelet-neutrophil aggregates (PNA) in stimulated whole blood. Correlations between the percentage of monocytes that were positive for platelet marker CD42b (platelet-monocyte aggregates, PMA) and the percentage of CD14- neutrophils that were positive for platelet marker CD42b (PNA) in whole blood following stimulation with (a) 0.3µM ADP, (b) 3µM ADP, (c) 1µM TRAP-6, and (d) 10µM TRAP-6. Data from patients with TB (red circles), symptomatic respiratory patients (blue circles), and healthy controls (black circles). *r* calculated using Spearman’s non-parametric coefficient of correlation.
(TIF)

S4 Fig Correlation between platelet P-selectin expression and platelet-monocyte aggregation (PMA) in stimulated whole blood. Correlations between the percentage of platelets that were positive for platelet activation marker P-selectin and the percentage of monocytes that were positive for platelet marker CD42b (platelet-monocyte aggregates, PMA) in whole blood following stimulation with (a) 0.3µM ADP, (b) 3µM ADP, (c) 1µM TRAP-6, and (d) 10µM TRAP-6. Data from patients with TB (red circles), symptomatic respiratory patients (blue circles), and healthy controls (black circles). *r* calculated using Spearman’s non-parametric coefficient of correlation.
(TIF)

S5 Fig Correlation between platelet P-selectin expression and platelet-neutrophil aggregates (PNA) in stimulated whole blood. Correlations between the percentage of platelets that were positive for platelet activation marker P-selectin and the percentage of CD14- neutrophils that were positive for platelet marker CD42b (PNA) in whole blood following stimulation with (a) 0.3µM ADP, (b) 3µM ADP, (c) 1µM TRAP-6, and (d) 10µM TRAP-6. Data from patients with TB (red circles), symptomatic respiratory patients (blue circles), and healthy controls (black circles). *r* calculated using Spearman’s non-parametric coefficient of correlation.
(TIF)

S6 Fig Correlation between platelet GPIIb/IIIa expression and platelet-monocyte aggregates in stimulated whole blood. Correlations between the percentage of platelets that were positive for platelet activation marker GPIIb/IIIa and platelet-monocyte aggregates (PMA) in whole blood following stimulation with (a) 0.3µM ADP, (b) 3µM ADP, (c) 1µM TRAP-6, and (d) 10µM TRAP-6. Data from patients with TB (red circles), symptomatic respiratory patients (blue circles), and healthy controls (black circles). *r* calculated using Spearman’s non-parametric coefficient of correlation.
(TIF)

S7 Fig Correlation between platelet GPIIb/IIIa expression and platelet-neutrophil aggregates in stimulated whole blood. Correlations between the percentage of platelets that were positive for platelet activation marker GPIIb/IIIa and platelet-neutrophil aggregates (PNA) in whole blood following stimulation with (a) 0.3µM ADP, (b) 3µM ADP, (c) 1µM TRAP-6, and (d) 10µM TRAP-6. Data from patients with TB (red circles), symptomatic respiratory patients (blue circles), and healthy controls (black circles). *r* calculated using Spearman’s non-parametric coefficient of correlation.
(TIF)

S8 Fig Correlation between platelet P-selectin expression and platelet GPIIb/IIIa expression in stimulated whole blood. Correlations between the percentage of platelets that were positive for platelet activation markers P-selectin and GPIIb/IIIa in whole blood following stimulation with (a) 0.3µM ADP, (b) 3µM ADP, (c) 1µM TRAP-6, and (d) 10µM TRAP-6. Data from patients with TB (red circles), symptomatic respiratory patients (blue circles), and healthy controls (black circles). *r* calculated using Spearman’s non-parametric coefficient of correlation.
(TIF)

S1 Data This file contains the complete raw experimental data supporting the findings of this study. Data are presented in their original, unprocessed form as recorded during each experiment. Individual worksheets correspond to specific figures presented in the main manuscript and are labelled accordingly, and datasets include all relevant replicate measurements and control conditions.
(XLSX)
