# Supplementary material for: Platelet-leucocyte interactions drive MMP-mediated tissue damage in tuberculosis
Source: PLoS Pathog. 2026 May 11;22(5):e1014205. doi: 10.1371/journal.ppat.1014205 (PMC13229373; doi:10.1371/journal.ppat.1014205)
Supplement: S1 Table — (DOCX) [file ppat.1014205.s001.docx]

S1 Table

| **Study code** | **Age/ sex** | **Bronchoscopy findings** | **Radiological findings** | **Auramine microscopy** | **MODS culture** | **Other tests positive for TB** | **Anti-TB treatment initiated** | **Days from procedure to treatment initiation** | **Diagnosis** |
| --- | --- | --- | --- | --- | --- | --- | --- | --- | --- |
| TBP-401 | 76F | Signs of acute inflammation | Chest radiograph showed interstitial alveolar infiltrate and tree-in-bud, left lower lobe. | Negative | Negative | No | No | N/A | COPD exacerbation |
| TBP-402 | 50M | Signs of acute inflammation | CT thorax normal. | Negative | Negative | No | No | N/A | No diagnosis |
| TBP-403 | 24M | Signs of acute inflammation | Chest radiograph showed tree-in-bud changes in left lower lobe and 6th lung segment | Negative | Positive | Positive GeneXpert | Yes | 2 | Pulmonary TB |
| TBP-404 | 54F | Abundant white secretions in right lung, and yellow-white secretions in left lower lobe. Signs of acute inflammation | Chest radiograph showed patchy alveolar changes in segments 3 and 4 | Negative | Negative | No | No | N/A | Bacterial pneumonia |
| TBP-405 | 87F | No report available. | No report available. | Negative | Negative | No | No | N/A | Squamous cell carcinoma of the lung |
| TBP-406 | 76F | Dense secretions in left lung. Signs of acute vs chronic inflammation | Chest radiograph showed bilateral interstitial shadowing and right sided opacity, ground glass changes, bilateral basal lung cysts. | Negative | Negative | No | No | N/A | Hypersensitivity pneumonitis |
| TBP-407 | 22M | Moderate secretions in left lung. Signs of chronic inflammation. | No report available. | Positive | Positive | No | Yes | 25 | Pulmonary TB |
| TBP-408 | 71M | Abundant yellow and white secretions in right lung. Signs of chronic inflammation. Exclude gastro-oesophageal reflux vs pulmonary TB. | Chest radiograph showed right apical cavity | Positive | Positive | No | Yes | 1 | Pulmonary TB |
| TBP-409 | 19F | Right lung: serosanguinous secretions. Signs of acute and chronic inflammation. | Chest radiograph showed bilateral apical opacification | Positive | Positive | Positive sputum culture | Yes | 1 | Pulmonary TB |
| TBP-410 | 54F | Erythematous mucosa, stomach contents and biliary yellow secretions in both lungs. Signs of acute inflammation. | Chest radiograph showed left cardiac border silhouette sign | Negative | Negative | No | No | N/A | Aspiration pneumonia |
| TBP-411 | 22F | Acute inflammation | Chest radiograph showed thick-walled cavity in right hemithorax with surrounding tree-in-bud changes | Negative | Negative | No | Yes | 7 | Pulmonary TB |
| TBP-412 | 21M | Acute inflammation | Chest radiograph showed alveolar pattern in right inferior lobe. | Negative | Negative | No | No | N/A | No diagnosis |
| TBP-413 | 70M | Acute inflammation | Chest radiograph showed bronchiectasis in right superior lobe and left superior lobe, fibrotic changes. | Negative | Negative | No | No | N/A | Bronchiectasis. |
| TBP-414 | 25M | No report available. | No report available. | Pauci-bacillary | Positive | Positive culture at HNCH | Yes | 4 | Pulmonary TB |
| TBP-415 | 64F | Acute inflammation | No report available. | Negative | Negative | No | No | N/A | Lung cancer |
| TBP-417 | 19F | Chronic inflammation | Chest radiograph showed right apical infiltration. | Negative | Negative | No | Yes | 2 | Pulmonary TB |
| TBP-418 | 45M | Erythematous mucosa, reduction in bronchial diameter, and mucoid secretions. Chronic and acute inflammation. | CT chest showed nodular lesions in left basal segments. | Negative | Negative | No | No | N/A | Hypersensitivity pneumonitis |
| TBP-419 | 51F | No report available. | No report available. | Negative | Negative | No | No | N/A | Chronic bronchitis |
| TBP-420 | 73F | No report available. | No report available. | Negative | Negative | No | No | N/A | Chronic inflammation secondary to gastric reflux. |
| TBP-421 | 62M | Frothy mucoid secretions in right upper lobe, whitish secretions in left lobe. Chronic inflammation. | Chest radiograph showed diffuse interstitial shadowing. Lesion in left lower lobe, possibly consistent with abscess, and extending into left apex. | Pauci-bacillary | Indeterminate | No | Yes | Not known – patient lost to follow-up | Pulmonary TB |
| TBP-422 | 66M | Abundant mucoid secretions in right lung and left lower lobe. Acute inflammation. | Chest radiograph showed interstitial changes, with ground glass, predominantly in inferior lobes. | Negative | Negative | No | No | N/A | Interstitial lung disease. |
| TBP-423 | 20F | Acute inflammation. | Chest radiograph showed extensive cavitation in left and right apices. | Negative | Negative | No | Yes | 11 | Pulmonary TB |
| TBP-424 | 60F | Mucoid secretions in both lungs. Chronic bronchial inflammation. | Chest radiograph showed right apical opacity with mild infiltration in the left upper lobe. | Negative | Negative | No | No | N/A | Chronic allergic bronchitis |
| TBP-425 | 35F | No report available. | No report available. | Negative | Positive | BAL GeneXpert positive | Yes | 2 | Pulmonary TB |
